# Uncertainty-aware deep-learning model for prediction of supratentorial hematoma expansion from admission non-contrast head computed tomography scan

**DOI:** 10.1038/s41746-024-01007-w

**Published:** 2024-02-06

**Authors:** Anh T. Tran, Tal Zeevi, Stefan P. Haider, Gaby Abou Karam, Elisa R. Berson, Hishan Tharmaseelan, Adnan I. Qureshi, Pina C. Sanelli, David J. Werring, Ajay Malhotra, Nils H. Petersen, Adam de Havenon, Guido J. Falcone, Kevin N. Sheth, Seyedmehdi Payabvash

**Affiliations:** 1 Department of Radiology and Biomedical Imaging, Yale School of Medicine, New Haven, CT USA; 2 Department of Otorhinolaryngology, University Hospital of Ludwig Maximilians Universität München, Munich, Germany; 3 Stroke Institute and Department of Neurology, University of Missouri, Columbia, MO USA; 4 Department of Radiology, Northwell Health, Manhasset, NY USA; 5 Stroke Research Centre, University College London, Queen Square Institute of Neurology, London, UK; 6 Department of Neurology, Yale School of Medicine, New Haven, CT USA

**Keywords:** Brain imaging, Stroke

## Abstract

Hematoma expansion (HE) is a modifiable risk factor and a potential treatment target in patients with intracerebral hemorrhage (ICH). We aimed to train and validate deep-learning models for high-confidence prediction of supratentorial ICH expansion, based on admission non-contrast head Computed Tomography (CT). Applying Monte Carlo dropout and entropy of deep-learning model predictions, we estimated the model uncertainty and identified patients at high risk of HE with high confidence. Using the receiver operating characteristics area under the curve (AUC), we compared the deep-learning model prediction performance with multivariable models based on visual markers of HE determined by expert reviewers. We randomly split a multicentric dataset of patients (4-to-1) into training/cross-validation (*n* = 634) versus test (*n* = 159) cohorts. We trained and tested separate models for prediction of ≥6 mL and ≥3 mL ICH expansion. The deep-learning models achieved an AUC = 0.81 for high-confidence prediction of HE_≥6 mL_ and AUC = 0.80 for prediction of HE_≥3 mL_, which were higher than visual maker models AUC = 0.69 for HE_≥6 mL_ (*p* = 0.036) and AUC = 0.68 for HE_≥3 mL_ (*p* = 0.043). Our results show that fully automated deep-learning models can identify patients at risk of supratentorial ICH expansion based on admission non-contrast head CT, with high confidence, and more accurately than benchmark visual markers.

## Introduction

Hematoma expansion (HE) affects 13–38% of patients with acute intracerebral hemorrhage (ICH)^1,2^, and is an independent determinant of morbidity and mortality^3^. Every 1 mL increase in hematoma volume is associated with a 5% higher risk of death or long-term functional dependency^4^. As a modifiable predictor of outcome, HE is a potential target for anti-expansion interventions or hemostatic therapies^5^. Identification of patients at risk of HE for targeted therapy, can increase the chances of treatment benefit from anti-expansion interventions^6^. However, there is yet no reliable tool available for prediction of HE in acute ICH settings.

Non-contrast head Computed Tomography (CT)—as a fast and widely available imaging modality—is the first line of diagnosis in patients with suspected ICH. An actively hemorrhagic cerebral hematoma—at risk of HE—tends to contain a mixture of hyperdense acute and hypodense subacute clot materials, leading to a heterogenous appearance on head CT^7–10^. Many groups have described different visual markers of ICH heterogeneity pattern and irregular shape on admission non-contrast head CT, which are associated with subsequent HE—including swirl, hypodensity, black hole, blend, fluid level, island, and satellite signs^7–15^. Such visual markers are, however, subject to inter-reader variability and overlapping definitions, indicating a need for reliable neuroimaging tools for prediction of HE.

Deep-learning algorithms, which are specialized in image pattern recognition, can potentially address this unmet need by identifying CT imaging patterns associated with a higher risk of HE. Such automated image analysis tools can provide fast and accurate HE-risk stratification in acute ICH settings, with reproducible results across different centers^16^. Many groups have applied Convolutional Neural Networks (CNN) to detect ICH on non-contrast head CT^17–19^. However, there are only a few reports about HE prediction using CNN^20–22^. We aimed to train, optimize, and validate CNN-based models for end-to-end fully automated prediction of HE from head CT images. In addition, we implemented Monte Carlo dropout method to estimate prediction uncertainty and achieve high-confidence prediction of supratentorial ICH expansion^23^. We compared final deep-learning model performance with benchmark visual predictors of HE, which were based on expert review of scans (Supplementary Table 1).

## Results

### Patients’ demographics

**Fig. 1:**
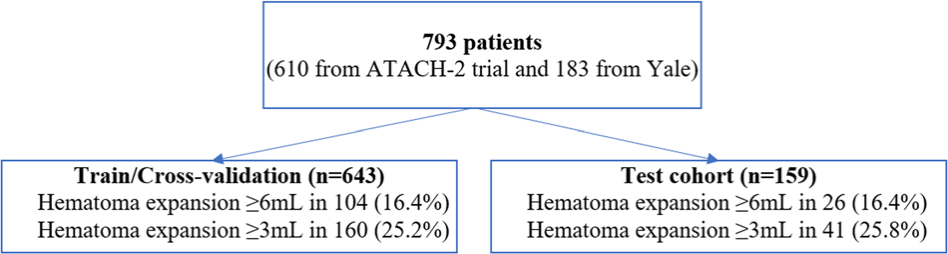
Patients’ flowchart. The dataset was split 4:1 into training/cross-validation (5-fold) and test cohort, which was isolated from the training process. ATACH Antihypertensive Treatment of Acute Cerebral Hemorrhage Trial, HE Hematoma Expansion.

A total of 793 patients (610 patients from Antihypertensive Treatment of Acute Cerebral Hemorrhage (ATACH-2) trial and 183 patients from Yale) were included in our analysis, and split (4-to-1) into 634 training/cross-validation and 159 test cohorts (Fig. 1). Table 1 summarizes the demographic characteristics, treatments, and baseline clinical information of the training/cross-validation and independent test cohorts. The rates of HE_≥6 mL_, and HE_≥3 mL_ were respectively 16%, and 25% in both training/cross-validation and test cohorts (Table 1). Supplementary Table 2 compares the clinical and demographic characteristics of the ATACH-2 and Yale datasets. Overall, the ATACH-2 dataset had higher rates of Asian patients, but lower rate of White patients, and smaller hematoma volumes compared to the Yale dataset.

**Table 1:** The demographic and clinical characteristics of patients in training/cross-validation versus test cohorts.

	Cross-validation/training data (*n* = 634)	Test data (*n* = 159)	*P* value
Hematoma expansion ≥6 mL—*n* (%)	104 (16.4%)	26 (16.4%)	0.987
Hematoma expansion ≥3 mL—*n* (%)	160 (25.2%)	41 (25.8%)	0.886
Sex [male]—*n* (%)	364 (57.4%)	96 (60.4%)	0.018
Age^a^ [years]—mean ± SD	63.4 ± 31.6	63.7 ± 34.3	0.780
Ethnic group—*n* (%)			
Hispanic	62 (9.8%)	16 (10.0%)	0.818
Not Hispanic	572 (90.2%)	143 (90.0%)	
Race—*n* (%)			
White	316 (49.9%)	66 (41.5%)	0.259
Black	113 (17.8%)	28 (17.6%)	
Asian	189 (29.8%)	56 (35.2%)	
Other	16 (2.5%)	9 (5.6%)	
Systolic blood pressure^a^ [mmHg] mean ± SD	171.4 ± 27.6	171.0 ± 25.2	0.867
History of hypertension—*n* (%)	514 (81.1%)	124 (78.0%)	0.389
History of diabetes mellitus type I/II—*n* (%)	148 (23.3%)	37 (23.2%)	1.000
History of hyperlipidemia—*n* (%)	235 (37.1%)	54 (34.0%)	0.359
History of atrial fibrillation—*n* (%)	62 (9.8%)	15 (9.5%)	<0.001
Glasgow Coma Scale score at baseline—*n* (%)			
3–11	105 (16.5%)	19 (11.9%)	0.010
12–14	164 (25.8%)	56 (35.2%)	
15	356 (56.2%)	84 (52.9%)	
unknown	1 (0.1%)	0	
NIH Stroke Scale score at baseline—*n* (%)			
0–4	146 (23.0%)	31 (19.5%)	<0.001
5–9	139 (21.9%)	47 (29.5%)	
10–14	149 (23.5%)	38 (23.9%)	
15–19	104 (16.4%)	27 (17.0%)	
20–25	71 (11.2%)	11 (6.9%)	
>25	23 (3.6%)	5 (3.2%)	
unknown	2 (0.4%)	0	
Baseline hematoma volume^a^ [mL]—mean ± SD	15.56 ± 16.88	14.41 ± 13.76	0.673
Follow-up hematoma volume^a^ [mL]—mean ± SD	18.03 ± 19.76	18.39 ± 19.86	0.618
CT			
Slice thickness^a^ [mm]—mean ± SD	4.5 ± 0.9	4.56 ± 0.91	0.673
In-plane pixel spacing^a^ [mm]—mean ± SD	0.458 ± 0.032	0.461 ± 0.031	0.726
Min axial image matrix [*n* × *n*]	418 × 418	512 × 512	
Max axial matrix [*n* × *n*]	512 × 734	512 × 666	
Number of slices—mean ± SD	37.3 ± 19.7	37.5 ± 16.5	

^a^Using two-sample *t* tests; others using the chi-square test.

*SD* standard deviaton.

### Automated hematoma segmentation

**Fig. 2:**
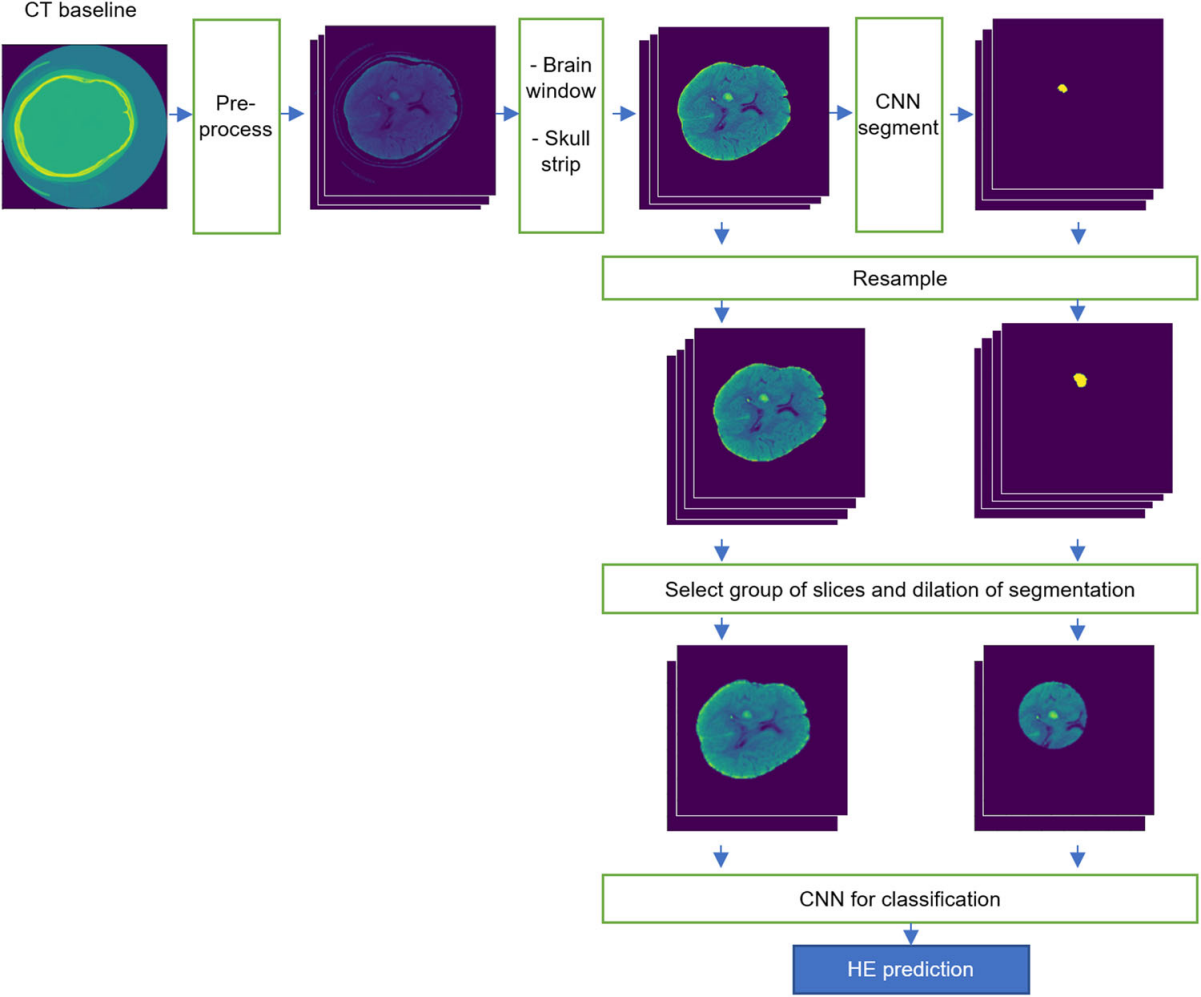
Fully automated pipeline for prediction of hematoma expansion. As detailed in the methods, we first applied voxel value constraint and morphology-based skull stripping to remove the bony calvarium. Then, we applied a U-Net based convolutional neural network (CNN) to segment hematoma lesions, followed by resampling to 1 mm isotropic space. Next, we cropped all brain scans to fixed-size boxes centered around the hematoma lesion. The dual input for the optimal deep-learning model (Table 2) included both the cropped box of axial head CT and the dilated mask of hematoma and surrounding tissue. The CNN model structure is provided in our GitHub and Supplementary Fig. 2.

We developed and tested automated hematoma segmentation models using similar training/validation and test data splitting. In the 5-fold cross-validation, the best segmentation algorithm achieved an averaged Dice similarity coefficient (DSC) of 0.86 ± 0.01, and volume similarity (VS) of 0.91 ± 0.01 in validation folds. This model achieved a DSC of 0.87, and VS of 0.91 in the test cohort. This segmentation model generated all automated hematoma masks, which were then dilated to provide additional inputs to axial head CT slices for the HE prediction model (Fig. 2).

### Development and testing of deep-learning models for prediction of HE

Table 2 summarizes the performance of HE prediction models with different inputs in the testing cohorts using DenseNet121^24^ as the backbone CNN. The best results were achieved with dual inputs from head CT axial slices and dilated mask of automatically segmented hematoma (Table 2 and Fig. 2).

**Table 2:** Predictive performance of deep-learning models with different inputs.

	Metrics		
	AUC	Sensitivity	Specificity
Input			
Prediction of ≥6 mL hematoma expansion			
CT slices	0.72	0.42	0.93
CT slices + hematoma mask	0.76	0.65	0.75
CT slices + dilated hematoma mask	0.79	0.62	0.84
Monte Carlo Dropout excluding uncertain patients (input = CT slices + dilated hematoma mask)	**0.81**	**0.62**	**0.81**
Prediction of ≥3 mL hematoma expansion			
CT slices	0.72	0.51	0.84
CT slices + hematoma mask	0.75	0.56	0.85
CT slices + dilated hematoma mask	0.75	0.56	0.82
Monte Carlo Dropout excluding uncertain patients (input = CT slices + dilated hematoma mask)	**0.80**	**0.84**	**0.61**

Summary of model prediction performance in the test cohort with different inputs. Our final model was based on DenseNet121 3D convolutional neural network (CNN) with inputs from axial slices encompassing the hematoma in addition to dilated circular mask of automatically segmented hematoma lesions—which included peri-hematomal parenchyma (Fig. 2).

*AUC* area under the curve (of receiver operating characteristics analysis).

The performance metrics of the final model are depicted in bold font.

For HE_≥6 mL_, the average AUC, sensitivity, and specificity in five validation folds of cross-validation were 0.73 ± 0.09, 0.65 ± 0.08, and 0.75 ± 0.08 respectively: with the best-performing model achieving an AUC = 0.82, sensitivity = 0.62, and specificity = 0.86 in the validation fold. In the independent test cohort, our model achieved an AUC (95% Confidence Interval) of 0.80 (0.71–0.90), sensitivity = 0.77, and specificity = 0.80. After excluding patients with high uncertainty in model prediction (8 out of 159), the final deep-learning model predicts HE_≥6 mL_ with AUC = 0.81 (0.70–0.92), sensitivity = 0.62, and specificity = 0.81.

For HE_≥3 mL_, the average AUC, sensitivity, and specificity across five validation folds in were 0.73 ± 0.05, 0.62 ± 0.15, and 0.78 ± 0.05, respectively: with the best-performing model achieving an AUC = 0.81, sensitivity = 0.71, and specificity = 0.76 in the validation fold. In the independent test cohort, our model achieved an AUC = 0.75 (0.67–0.84), sensitivity = 0.56, and specificity = 0.82. After excluding patients with high uncertainty in model prediction (16 out of 159), the final deep-learning model predicts HE_≥3 mL_ with an AUC = 0.80 (0.71–0.88), sensitivity = 0.84, and specificity = 0.61.

**Fig. 3:**
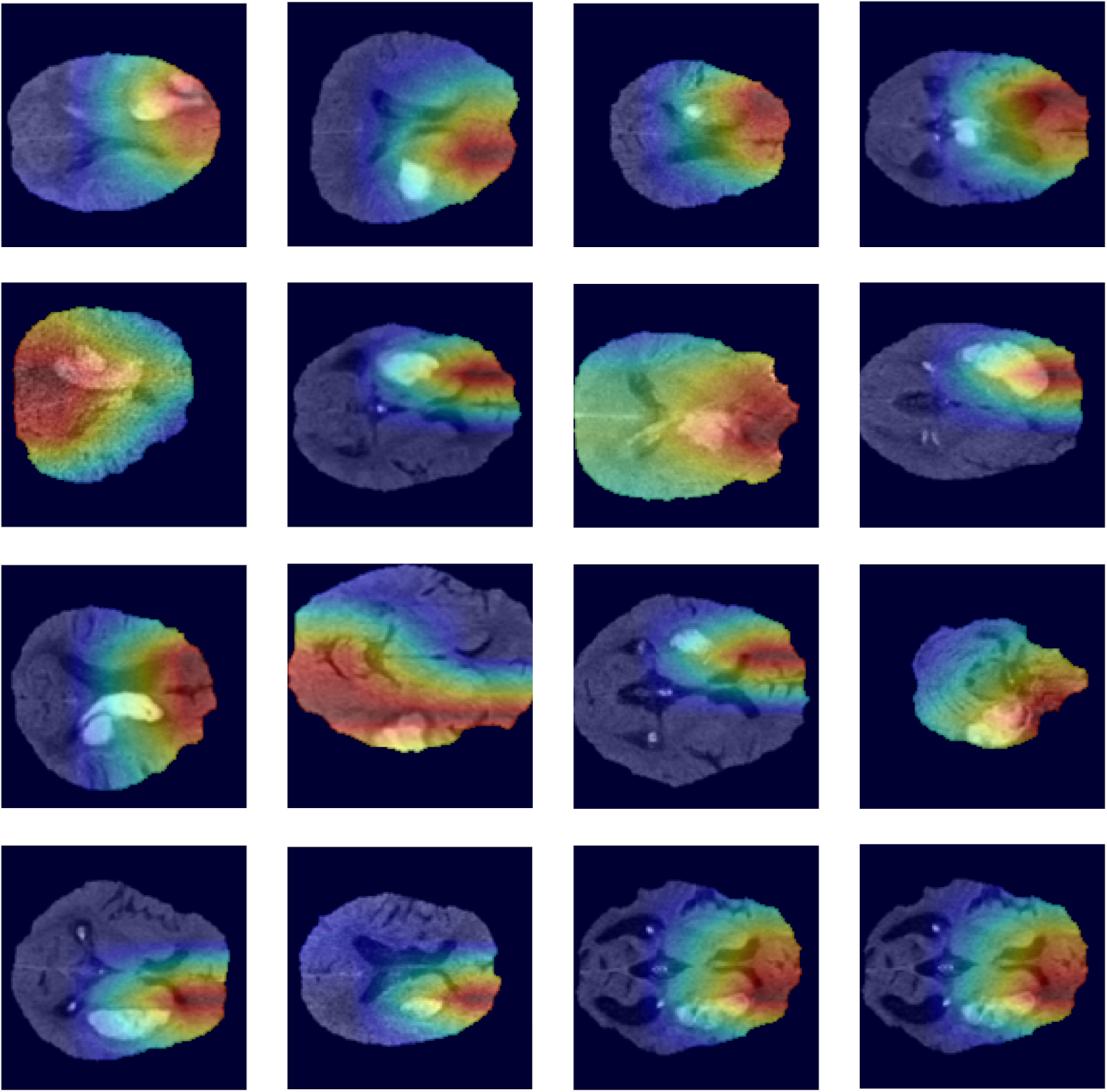
Attention maps for visual validation and interpretation of deep-learning model performance. Examples of 3D attention maps highlighting the brain regions with the highest impact on decisions of deep-learning models confirm that predictions of hematoma expansion were based on imaging patterns of parenchymal hemorrhage and surrounding tissues^25^.

Supplementary Table 3 represents the confusion matrix for all models; and supplementary Table 4 summarizes precision, accuracy, and Matthew’s correlation coefficients of the models. The heatmaps confirmed that models’ decisions were predominantly based on attention to ICH and surrounding parenchyma on head CTs, as high-impact regions had an overlap with dilated ICH masks in all subjects (Fig. 3)^25^. All the codes are publicly available on GitHub (https://github.com/anhtrnyaleedu/HE).

### Comparison of deep-learning model with visual markers of HE

**Fig. 4:**
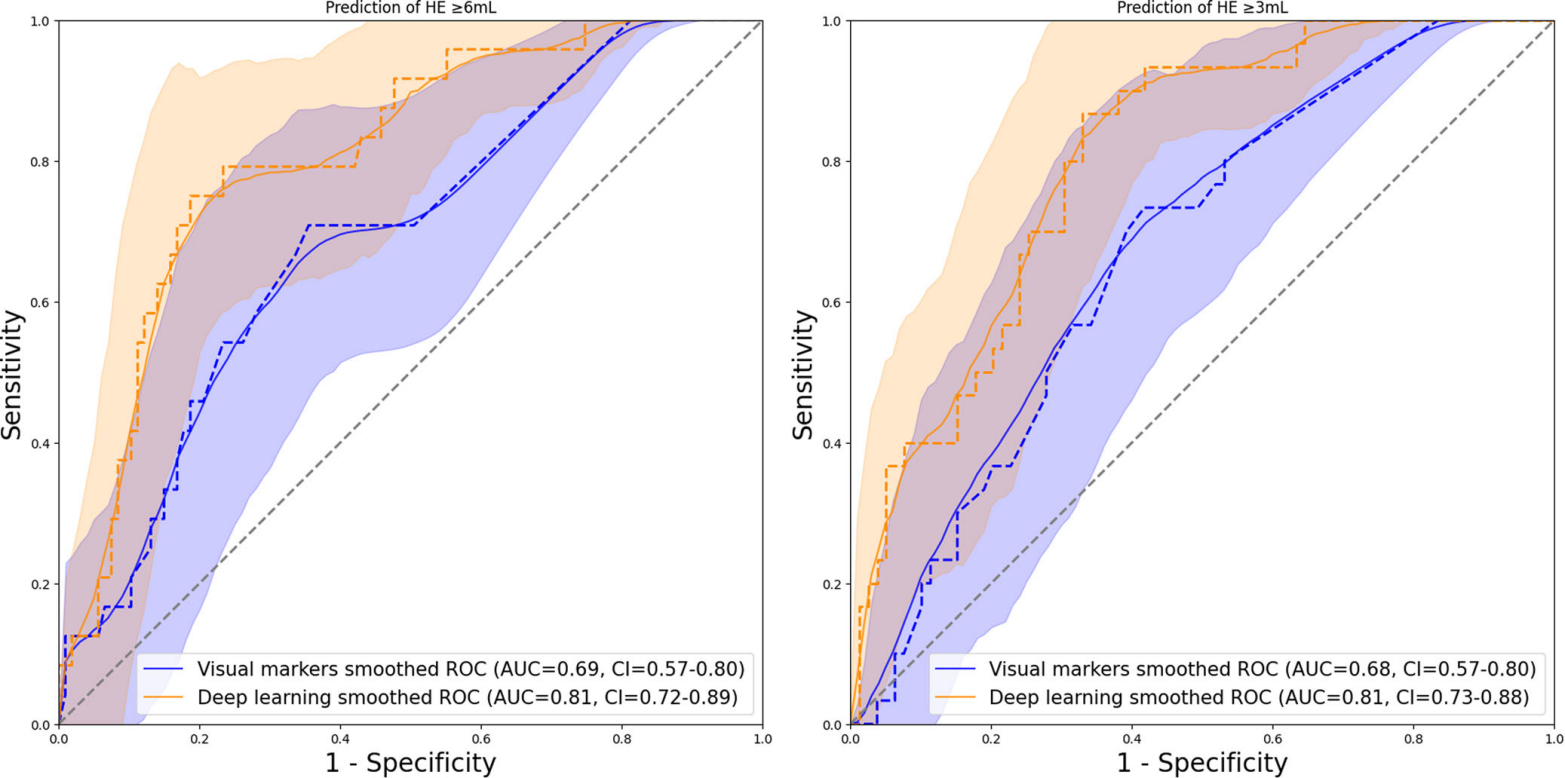
Comparison of predictive performance of deep-learning versus visual-marker models. The raw and smoothed (95% confidence interval) area under the curve (AUC) of receiver operating characteristic for high-confidence prediction of ≥6 mL (left panel) and ≥3 mL (right panel) hematoma expansion (HE) by the deep-learning model (red) versus visual-marker model (blue). The deep-learning model had higher AUC than visual markers in prediction of HE_≥6 mL_ (*p* = 0.036) and HE_≥3 mL_ (*p* = 0.043).

**Fig. 5:**
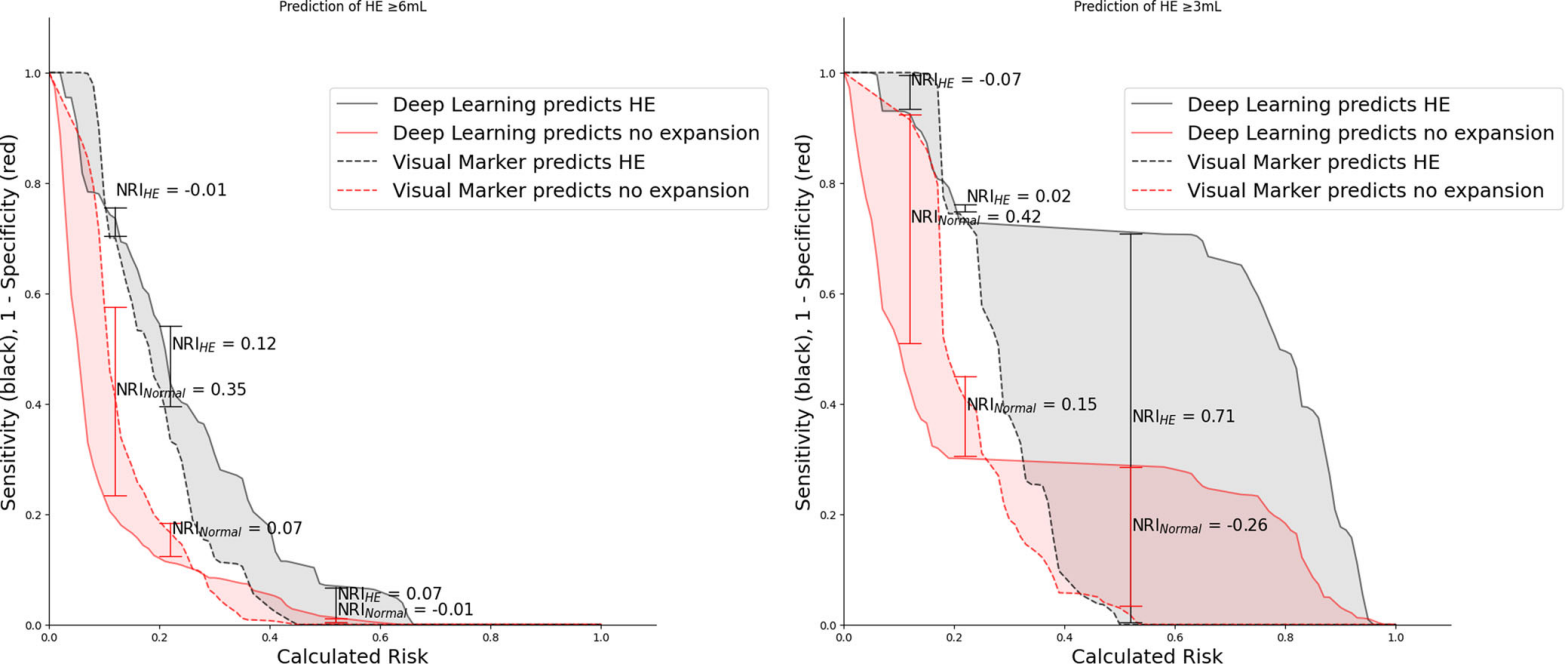
Comparison of HE-risk assessment plot between the deep-learning and visual-marker models. The risk assessment plots for prediction of hematoma expansion (HE, in black) versus non-expansion (red) show that deep-learning (solid line) models were more sensitive than visual-marker models (dashed lines) in identification of patients at risk of both HE_≥6 mL_ (left panel) and HE_≥3 mL_ (right panel). The deep-learning model improved HE_≥6 mL_ risk assessment (left panel) with an NRI (Net Reclassification Index) of 0.69 (0.28–1.10) (*p* < 0.001), and an Integrated Discrimination Improvement (IDI) of 0.1073 (0.024–0.190) (*p* < 0.001). In addition, the deep-learning model improved HE_≥3 mL_ risk assessment with an NRI of 0.75 (0.39–1.09) (*p* < 0.001) and an IDI of 0.2307 (0.12–0.48) (*p* < 0.001). Risk assessment plots demonstrate that deep-learning models increased sensitivity and identified more at-risk patients for HE compared to visual markers.

Supplementary Table 5 summarizes the distribution of eight visual predictors of HE between the train/cross-validation and independent test cohorts. The inter-rater agreement ranged from 0.44 to 0.61 (similar to our previous reports)^26^. After fitting logistic regression models for prediction of HE in the training/cross-validation cohort based on combination of the visual markers, we compared the predictive performance of visual-marker models with high-confidence predictions of deep-learning models in the test cohort. The visual-marker model achieved an AUC = 0.69 (0.58–0.80), sensitivity = 0.50, and specificity = 0.80 for prediction of HE_≥6 mL_, and an AUC = 0.68 (0.58–0.79), sensitivity = 0.63, and specificity = 0.70 for prediction of HE_≥3 mL_. Deep-learning models achieved higher AUCs compared to visual-marker models for predicting HE_≥6 mL_ (*p* = 0.036) and HE_≥3 mL_ (*p* = 0.043)—the ROC curves are depicted in Fig. 4. Additional risk assessment plots (Fig. 5) also demonstrate that deep-learning models increased sensitivity and identified more at-risk patients for HE compared to visual markers. Of note, the baseline hematoma volume alone could predict HE_≥6 mL_ with an AUC = 0.59, sensitivity = 0.42, and specificity = 0.81; and HE_≥3 mL_ with AUC = 0.62, sensitivity = 0.52, and specificity = 0.67. The deep-learning models had higher AUC compared to hematoma volume for prediction of either HE_≥6 mL_ (*p* = 0.004) or HE_≥3 mL_ (*p* = 0.004).

### Association of HE predictions with poor outcomes and death

Among patients with high-confidence prediction of HE_≥6 mL_, the odds ratios of poor outcome and death were 2.92 (*p* = 0.017), and 6.47 (*p* < 0.001), respectively. Among patients with high-confidence prediction of HE_≥3 mL_, the odds ratios of poor outcome and death were 1.69 (p = 0.18), and 5.70 (*p* = 0.001), respectively. In the same cohorts, patients with (ground truth) HE had higher odds of poor outcomes and death. For HE_≥6 mL_, the odds ratios of poor outcome and death were 2.48 (*p* = 0.07), and 4.38 (*p* = 0.006); and for HE_≥3 mL_, the odds ratios of poor outcome and death, were 2.47 (*p* = 0.02), and 2.42 (*p* = 0.13), respectively.

## Discussion

We developed, optimized, and validated fully automated uncertainty-aware deep-learning models for high-confidence prediction of supratentorial ICH expansion based on admission non-contrast head CT scans. We found that a dual input for DenseNet121 CNN with axial slices and dilated mask of (automatically segmented) hematomas produce optimal predictions. We used Monte Carlo dropout to generate estimates of the deep-learning model prediction uncertainty for each patient and used that to identify subset of patients with high-confidence prediction^23^. Compared to multivariable models combining eight benchmark visual predictors of HE, the deep-learning models had higher accuracy and improved risk assessment. Nevertheless, an automated model offers several advantages over visual assessment of CT scans including timely prediction in acute ICH settings, reduced dependence on local expertize for interpretation of head CTs, and reproducible results across different centers. Such deep-learning models can quickly and automatically identify those patients who are most likely to benefit from anti-expansion therapies, and potentially guide treatment decisions in acute ICH settings.

Different cut-offs have been proposed for binary categorization of HE^27–29^. Dowlatshahi et al.^27^ reported that ≥26%, ≥33%, ≥3 mL, ≥6 mL, and ≥12.6 mL HE are associated with 2.59, 2.73, 2.99, 3.11, and 3.98 odds ratio for poor outcome, respectively^27^. Recently, many groups have adopted an increase in hematoma size of “≥33% or ≥6 mL” as HE definition^29^. However, we noted that 33% increase in hematoma volume for HE prediction was prone to inter-rater variability: for example, in a patient with a 1-mL ICH at admission, a 0.1 mL difference between hypothetical 1.3 versus 1.4 mL hematoma segmentation on follow-up scan would change HE binary classification. Thus, we decided to develop and validate models for HE_≥6 mL_ as well as HE_≥3 mL_. In our cohort, both HE_≥6 mL_ and HE_≥3 mL_ were associated with higher odds of poor outcomes and death during 3-month follow-up. Notably, those identified at risk of HE by the deep-learning model also had higher odds of poor outcome and death, highlighting the clinical relevance of models’ predictions.

Some of the prior studies applying deep-learning for prediction of HE were limited by smaller sample sizes^20,21^. In the largest sample size thus far, Teng et al.^22^ utilized a dataset of 3016 ICH patients (20% with HE) to train and validate a deep-learning model for prediction of HE_≥6 mL_. In an independent test set of *n* = 118 ICH patients (24% with HE), their model achieved 89.3% sensitivity, 77.8% specificity, and a Yoden index of 0.671. They compared the model with BAT score, which is based on blend sing, hypodensity, and CT time gap from the onset.

Overall, our study has several advantages over prior reports: utilization of a large multicentric multinational dataset, reporting improved prediction accuracy by dual-input model design, implementation of prediction uncertainty estimates to identify patients with high-confidence prediction, direct comparison of deep-learning model with benchmark visual predictors in an independent test cohort, and evaluating the risk assessment benefits of deep-learning model over benchmark visual predictors.

In recent years, there has been increasing recognition of the need for quantifying confidence and uncertainty of predictions by artificial intelligence applications in medical field^30,31^. Since the primary outputs of deep-learning classifiers are not necessarily calibrated, they should not be assumed as empirical probabilities without attention to prediction uncertainty^32^. Different methods and metrics have been proposed to estimate uncertainty of a deep-learning model prediction^33^. In this study, we applied a recently described method, which involves Monte Carlo dropout to randomly drop a proportion of nodes within the model architecture when generating predictions^23^. Thus a single input sample undergoes multiple forward passes through different (suboptimal) variations of the final optimized network (with dropped nodes) and generates a distribution of predictions during the inference step^34^. Then, we applied Shannon’s entropy, which measures the randomness in the distribution of model prediction outputs^35^. The optimal entropy cutoff point was a tradeoff between model prediction accuracy and percentage of patients excluded due to uncertainty. Our model was able to predict both ≥6 mL and ≥3 mL supratentorial HE with high confidence and accuracy (AUC > 0.8) in >90% of patients in the independent test set. The deep-learning model predictions were more accurate than benchmark visual predictors of HE and were associated with higher likelihood of poor outcome and death.

The use of dual input for model design was supported by prior studies, our multistep experiments, and pathophysiology of HE. Prior radiomics studies have shown the relevance of hematoma lesion texture features on head CT in prediction of HE^26,36–38^. Teng et al. also combined CNN and radiomics features extracted from ICH lesions to predict HE^22^. From a technical standpoint, given the small and imbalanced number of CT slices that contain hematoma lesions, targeting the contiguous stack of slices containing ICH can theoretically improve the CNN prediction by removing the noise and inefficient features from large number of slices without any hematoma. In addition, given the imperfection of automated segmentation, a dilated mask can ensure inclusion of all slices and brain regions with hemorrhage. The inclusion of parenchyma around the ICH can also provide neurobiologically relevant information from peri-hematomal edema, which contributes to pathogenesis of HE^39^. As summarized in Table 2, we found an incremental improvement in prediction accuracy by addition of dual input from hematoma lesion mask to axial head CT slices, and then from dilated circular mask including hematoma and surrounding tissues (Supplementary Fig. 3). Our final model, combined broad features from consecutive axial slices of skull-stripped head CT, centered around the ICH with focal features from dilated masks containing ICH and surrounding parenchyma for the prediction of HE (Fig. 2 and Supplementary Fig. 2).

While different visual markers of ICH on admission head CT are reported in association with increased risk of HE^7–15^, each marker independently, and in combination with each other, have limited predictive accuracy^40,41^. Some authors reported 0.70 to 0.96 inter-rater agreement in evaluation of these markers^41,42^, which was higher than ours^26^, and reflects the inter-institutional variability in application of these markers. Nevertheless, these visual markers currently serve as benchmark tools available to predict HE based on admission non-contrast head CT in patients with acute ICH^11^. In our series, deep-learning models had higher accuracy and improved risk stratification compared to combination of eight different visual markers for predicting HE_≥6 mL_ and HE_≥3 mL_. Baseline hematoma volume is also a main predictor of HE;^43^ however, we showed that deep-learning models had higher AUC than hematoma volume in predicting HE. Thus, the predictive performance of the model is not solely due to its estimate of baseline ICH volume. Overall, our deep-learning model could provide high-confidence and more accurate prediction of supratentorial HE compared to visual predictors and baseline hematoma volume.

In this study, we complemented AUC analysis with Net Reclassification Improvement (NRI) and Integrated Discrimination Improvement (IDI) indices, which provide additional insights to how new models may improve risk stratification compared to an existing one^44^. While the AUC is a valuable metric, it alone may not offer a complete picture for a comprehensive analysis of the impact of new biomarkers and models. Overall, deep-learning models were more sensitive in the identification of patients at risk of HE, with a net improvement in risk assessment compared to visual markers (Fig. 5). However, NRI and IDI metrics have been criticized for overfitting, and such results should be interpreted with caution^45,46^.

Although our results are promising, the current study has several limitations. First, the study included only patients with primary supratentorial ICHs that were smaller than 60 mL. Notably, although for both ATACH-2 and Yale datasets, we applied similar baseline hematoma volume cutoff (<60 mL), the ATACH-2 trial patients had smaller ICH volumes (Supplementary Table 2). Given the hematoma volume differences, we combined the two datasets, since a model trained solely on ATACH-2 trial would predominantly be exposed to smaller baseline ICH data. Moreover, although ATACH-2 trial found no treatment benefit from intensive blood pressure reduction, follow-up exploratory analysis in subset of patients with basal ganglia ICH (444 of 1000)^47^, those receiving ultra-early treatment (within 2 h of onset, 354 patients)^48^, or those from Asia (*n* = 537)^49^ reported lower rates of HE in intensive treatment versus controls. In subgroup of 610 patients from ATACH-2 trial, who were included in our study, we could not replicate any of prior sub-cohort analysis showing treatment benefit in preventing either HE_≥6 mL_ or HE_≥3 mL_. This can be in part due to difference in the definitions of HE used in the trial (i.e. ≥33% or ≥6 mL)^50^ and/or hematoma volume measurements between our manual segmentation versus those from the clinical trial imaging core. Of note, there was no significant difference between admission systolic blood pressure between ATACH-2 versus Yale cohorts (Supplementary Table 2). In addition, reliable details of clinical course during the hospital stay were not available in all subjects; datasets with such detailed information can facilitate the development of clinically informed predictive models for identification of patients at risk of HE and neurological deterioration. Finally, while the datasets for our study were collected from multicenter hospitals and the model performance is superior to benchmark visual predictors of HE, it still needs to be externally validated in other institutes.

In summary, using a multicentric dataset of 793 patients with acute supratentorial ICH, we trained, optimized, and tested uncertainty-aware deep-learning models for high-confidence prediction of HE based on admission non-contrast head CTs. In independent test cohorts, the deep-learning models achieved higher accuracy, and improved the risk assessment, compared to a multivariable model combining eight benchmark visual predictors of HE. The multicentric nature of our training and validation datasets improves the stability and likely the generalizability of the final model. Such automated models have the potential to guide targeted treatment decisions in acute ICH settings.

## Methods

### Study design and participants

The clinical and imaging data for this study are from the Antihypertensive Treatment of Acute Cerebral Hemorrhage (ATACH-2) trial^50^, and the Yale Longitudinal Study of Acute Brain Injury^51^. ATACH-2 was a multicenter randomized trial enrolling 1000 patients who presented with a primary supratentorial ICH smaller than 60 mL, within 4.5 h from symptom onset and had systolic blood pressure above 180 mmHg, across 11 medical centers in United States, Germany, China, Taiwan, Japan, and South Korea (ClinicalTrials.gov ID NCT01176565)^50^. However, intensive blood pressure lowering had no treatment benefit in ATACH-2 trial^50^. We supplemented the ATACH-2 dataset with a patient cohort from the Yale Longitudinal Study of Acute Brain Injury, which has been prospectively collecting the longitudinal imaging and clinical information of patients presenting with acute brain injury (including spontaneous ICH) to the Yale health system^51^. From both datasets, we included adult patients (>18 years old) with acute supratentorial ICH who had admission non-contrast head CT and 24-hour follow-up scans, baseline hematoma volume <60 mL, and either high admission systolic blood pressure (>180 mmHg) or history of hypertension. We excluded patients who had head CT with axial slice thickness ≥5.8 mm, <28 slices, imaging artifacts affecting the hematoma lesion, any interval craniectomy or drain placement affecting the follow-up hematoma volume. This study received Institutional Review Board approval from all corresponding centers. Informed consent was waived given the retrospective nature of the study and the use of de-identified data.

### Ground truth segmentation and labeling of patients with and without HE

Using 3D-Slicer software^52^, trained research associates manually segmented hematoma lesions on all axial head CT slices from baseline and follow-up scans to calculate the ground truth hematoma volumes. Segmentations only included the intraparenchymal hemorrhage and excluded the intraventricular or extra-axial components. Then, all initial segmentations were further reviewed and edited by a neuroradiologist with over 10 years of experience. We used the manually segmented hematoma volumes on baseline and follow-up scans to define HE. The landmark study by Dowlatshahi et al.^27^ reported that HE of ≥3 mL and ≥6 mL have odds ratios of 2.99, and 3.11 in association with poor outcomes—defined by modified Rankin Scale (mRS) score 4–6^27^. Since the adoption of a percentage increase in hematoma volume (e.g., 33%) for HE prediction was prone to inter-rater variability—especially for smaller admission ICH volume, we decided to adopt ≥3 mL and ≥6 mL absolute increase in hematoma size for binary definitions of HE^28^.

### Image preprocessing for deep-learning models

#### Adjusting brain window-level in non-contrast head CT scan images

During visual inspection of medical imagery, radiologists usually apply window width and level settings to optimize the image contrast difference for evaluation of various tissue components^53^. In our study, we applied the brain window-level (level = 40, and width = 80) to optimize the image contrast between brain parenchyma and hemorrhage.

#### Skull stripping

To accentuate the model focus on brain parenchyma, we applied Hounsfield units (HU) restrictions followed by morphology-based methods to remove the skull from head CT images. Based on osseous structure density on non-contrast CT images, voxels with intensity <0 and >200 HU were removed to facilitate skull stripping^54,55^. Then, we applied morphology-based methods for skull stripping, including image dilation, erosion operations (binary_erosion(), remove_small_objects(), binary_dilation()), and removing boundary via the findContours() function.

#### Resampling of images

Head CT scans tend to differ in terms of voxel size and the number of slices across centers. Based on the patients’ image summary, we resized, cropped, or padded all 3D brain scans into a 512 × 512 × 48 matrix to achieve a consistent data size for the segmentation step. To maintain consistency of voxel spacing, we then resampled all images (and segmented masks) to isotropic 1-mm voxels (214, 214, 98)^56–58^.

#### Fixed-size cropped box containing axial slices centered around the hematoma

For more efficient computation, we generated fixed-size cropped boxes centered around the automatically segmented hematoma masks. After hematoma segmentation, the first slices containing hematoma mask were detected by traversing each scan from bottom to top, and the last slices containing hematoma mask were detected by moving backward. Thus, we could determine the contiguous slices that contained hematoma, and center a fixed-size crop box encompassing all slices with hematoma. Given the hematoma size and number of contiguous slices containing hematoma lesions, we transformed and resized the image to (192, 192, 96) and then further cropped the volume to the image center and a fixed-size box (128, 128, 96).

### Experiments design

Using a nested cross-validation scheme, we designed, trained, and validated different HE prediction models. The data from Antihypertensive Treatment of Acute Cerebral Hemorrhage (ATACH-2) trial and Yale Longitudinal Study of Acute Brain Injury were used in this study. Patients were randomly divided into 20% independent test data and 80% training data. Then, within the training cohort, we employed a 5-fold cross-validation method for both segmentation and classification tasks^59^. Thus, the training/cross-validation dataset was randomly divided into 5 parts, 4 of which were used for training, and the rest were used for validation, repeated ×5 times. All data splitting (train/validation/test) was performed using “Stratified K-Folds” splitting, preserving the percentage of samples for each class. All experiments were carried out by a computing device with AMD Ryzen 397SWX 32 Cores 2200H CPU (48 GB memory) and 4 GPUs (NVIDIA Quadro RTX 6000) with 32 GB memory, using Ubuntu operating system, Python 3.8, and the MONAI deep-learning framework^60^.

### Evaluation of model performance

#### Segmentation

DSC^61^ measures the volumetric overlap between segmentation results and ground truth. Dice is computed where A is the set of foreground voxels in the ground truth and B is the corresponding set of foreground voxels in the segmentation result.

1 $${Dice}=\frac{2(A\cap B)}{\left|A\right|+\left|B\right|}$$

Volume Similarity measures and compares the absolute volume of the segmented result and ground truth, defined as

2 $${VS}=1-\frac{\left|v1-v2\right|}{v1+v2}$$

#### Classification

Binary classifiers performance in imbalance data are routinely evaluated with Area Under the Curve (AUC) in Receiver Operating Characteristics (ROC) plots, sensitivity, and specificity^62^. Recall, also called sensitivity, is the proportion of true positives among all positives, and it varies between 0 and 1.

3 $${Sensitivity}={Recall}=\frac{{TP}}{{TP}+{FN}}$$

Specificity measures the proportion of true negatives that are correctly identified by the model.

4 $${Specificity}=\frac{{TN}}{{TN}+{FP}}$$

where TP = true positive, TN = true negative, FP = false positive, and FN = false negative.

The F1 score represents the harmonic mean of precision and recall, with its optimal value at 1 and its worst value at 0.

5 $$F1=\frac{2* ({Recall}* {Precision})}{({Recall}+{Precision})}$$

Matthews’s correlation coefficient (MCC) is a correlation coefficient between the ground truth and predicted in binary classifications with values between −1 and +1. A coefficient of +1 represents a perfect prediction, 0 no better than random prediction and −1 indicates total disagreement.

6 $${MCC}=\frac{\left({TP}* {TN}-{FP}* {FN}\right)}{\sqrt{\left({TP}+{FP}\right)\left({TP}+{FN}\right)\left({TN}+{FP}\right)\left({TN}+{FN}\right)}}$$

### Training a CNN model for automated hematoma segmentation

During our model development experiments, we evaluated head CT slices, segmented hematoma, dilated masks of hematoma, and their combinations as input for prediction model. We found that models with dual inputs from head CT slices and dilated masks encircling hematomas can achieve the best prediction performance. To devise a fully automated end-to-end predictive model, we incorporated an ICH segmentation pipeline to provide additional input for the prediction model. We trained and validated a 3D CNN model for automated ICH segmentation using SegResNet^63^, which is a deep semantic segmentation network based on the U-Net (details in the supplementary material). The input for the network has a size of 512 × 512 × 48 and the segmentation output is the intracerebral hemorrhage (ICH) region. During training, we randomly zeroed some of the elements of the input tensor with probability dropout_prob = 0.2. Manual segmentations of hematomas were used as gold standard (Supplementary Fig. 1). We used the DiceLoss as loss function, Adam as the optimizer^64^, CosineAnnealingLR as the learning rate scheduler, the sliding window inference method, and applied RandFlip for augmentation. We used both baseline and follow-up scans for training/validation and independent test data. Using stratified 4-to-1 splitting, we divided the dataset into training/cross-validation versus test cohorts (Fig. 1). We trained and optimized the model using a 5-fold cross-validation scheme in training/cross-validation cohort. Then, we trained the final model using hyperparameters from cross-validation on the whole training/cross-validation cohort and evaluated segmentation performance in the independent test cohort against manually segmented ICH masks as the ground truth. To evaluate the hematoma segmentation model performance, we used DSC and VS metrics, as described above.

### Training, validation, and testing of CNN models for prediction of HE

For the HE prediction model, we implemented a 5-fold cross-validation scheme with the same splits as the segmentation step described above. Then, we trained the final model in training/cross-validation data with hyperparameters from cross-validation to predict HE in the test cohort. Briefly, we trained a fully automated end-to-end model by combining hematoma segmentation and HE classification CNNs. In our experiments, we found that dual inputs from head CT slices and hematoma segmentation masks result in more accurate predictions. The hematoma mask from the automated segmentation step will serve as one of the two inputs for HE prediction CNN (Supplementary Fig. 2). For HE prediction, we implemented a 3D CNN with a binary classification output layer using our proposed method based on typical DenseNet121. Our optimal model had dual input from (1) consecutive axial CT slices centered around the ICH and (2) a dilated mask based on automated hematoma segmentation, which included both ICH and surrounding brain parenchyma. Applying binary_dilation() function, we dilated hematoma masks (Supplementary Fig. 3). For data augmentation, we used RandFlip (spatial_axis = 0, 1, and 2), RandZoom(min_zoom = 0.8, max_zoom = 1.2), RandRotate(range_x = (0.2, 0.2), range_y = (0.2, 0.2), range_z = (0.2, 0.2)), and RandAffine (shear_range = (0.2, 0.2)). Examples of augmented scans are depicted in Supplementary Fig. 4. We used (static) augmentation, creating ×4 times the number of data and balancing the data, before training the model on all training/cross-validation set together. Moreover, in each epoch, we applied additional (dynamic) augmentation during training process to make input slightly different in each epoch. To avoid overfitting, we used Dropout = 0.2, weight decay = 1e-5, and early stopping = 15 steps. Adam with learning rate = 0.001 was used as an optimization algorithm. The BCEWithLogitLoss, a binary cross-entropy loss that comes with a sigmoid function, was employed as a loss function (Supplementary Fig. 5). We applied StepLR learning scheduler to decrease learning rate until convergence with step_size = 15. The number of epoch = 100. The output is HE or not (1 or 0). The final prediction models had dual input from axial CT slices centered around the ICH and dilated masks from automated hematoma segmentation (Fig. 2 and Supplementary Fig. 2).

### Uncertainty-aware deep-learning model for high-confidence prediction^23^

The primary output of deep-learning models does not directly provide a measure of prediction confidence or (un)certainty. There are several metrics to estimate uncertainty of a deep-learning model prediction^33^. In this study, we applied Monte Carlo dropout and processes, which have recently been proposed for development of uncertainty-informed deep-learning models for high-confidence predictions in digital histopathology slides^23^. Commonly, dropout is only applied during the training of deep-learning models as a regularization method to avoid overfitting. Dolezal et al. have proposed using Monte Carlo dropout to generate a range of prediction probabilities and estimate deep-learning model uncertainty for high-confidence classification of lung adenocarcinoma versus squamous cell carcinoma on digital histopathology slides^23^. In this study, we adopted similar strategy to generate metrics of prediction uncertainty and achieve high-confidence prediction. After developing the optimal model, we deployed the Monte Carlo dropout (×100 times) when applying the model on the test set, using the enable_dropout() function with 0.2 dropout rate. In this method, the dropout generates (less than perfect) variations of the model by dropping some of the nodes of the trained model, and then applying them on the test set resulting in distribution of prediction probability values for each head CT in the test set. Then, we applied the Shannon entropy, which can provide a measure of uncertainty from such probability distribution^35^, with higher entropy representing higher prediction uncertainty. In this scheme, although lower entropy levels provide higher certainty, but they also exclude larger number of patients. Thus, the optimal cutoff needs to strike the proper balance between prediction accuracy/AUC, certainty/confidence in prediction, the number of patients excluded due to uncertainty, and the rate of HE in the remaining subjects. To achieve such optimal entropy threshold, one by one, we excluded the patient with the highest entropy (or the most uncertain prediction), and recalculated the AUC, accuracy, and rate of HE in remaining subjects. The process was stopped as soon as one of the metrics decreased from the initial test levels.

### Visual verification of deep-learning attention maps

To visually verify and confirm that the deep-learning model decisions were indeed based on imaging features of hemorrhage, we applied M3d-CAM^25^ to generate 3D attention maps and highlight brain regions with the highest impact on model prediction decisions on the original head CT. From the 3D input, we applied the Grad-Cam from M3d-CAM to extract the feature map from the last layer of the model, with the attention map resulting in 3D images. Then, we resized the attention map to scan dimension (e.g., 128 × 128 × 64), and overlaid the resized map onto the original scan.

### Prediction of HE based on CT imaging patterns determined by visual inspection

Applying the criteria by the international non-contrast CT ICH study group^11^, we determined eight visual predictors of HE on admission head CTs—namely, the blend sign, hypodensity, swirl sign, black hole sign, island sign, satellite sign, fluid level, and irregular shape (defined in supplementary Table 1)^26,36^. Trained research associates initially labeled admission head CTs, which were then reviewed, confirmed or corrected by a board-certified neuroradiologist (SP). Thus, each scan was reviewed by at least two raters. We applied logistic regression models for prediction of HE based on combining these visual markers in the training/cross-validation cohorts and then tested their prediction performance in independent test cohort.

### Comparing the CNN model with visual markers of HE—risk assessment plot

To compare the performance of the CNN model with current benchmark visual predictors of HE in the test cohort, we used the DeLong test for two related ROC curves. To measure the ability of the deep-learning model for risk assessment compared to a pre-existing visual marker^44^, we evaluated two additional metrics to determine whether deep-learning models could more accurately assess an individual patient’s risk for HE compared to visual markers. The Net Reclassification Improvement (NRI) estimates the proportion of patients reclassified to a more appropriate risk category and can be used in conjunction with the AUC^44^. The ${{NRI}}_{{event}}$ is the net proportion of patients with events reassigned to a higher-risk category and the ${{NRI}}_{{nonevent}}$ is the number of patients without events reassigned to a lower-risk category.

7 $${{NRI}}_{{event}}=P\left({up}|{event}\right)-P\left({down}|{event}\right)$$

8 $${{NRI}}_{{nonevent}}=P\left({down}|{nonevent}\right)-P\left({up}|{nonevent}\right)$$

9 $${{NRI}={NRI}}_{{event}}+{{NRI}}_{{nonevent}}$$

The IDI is a measure of the change in the discrimination slopes and shows the impact of new biomarkers on a binary predictive model. The IDI is the sum of the integrated sensitivity (IS) and integrated specificity (IP).

10 $${{IDI}}_{{event}}=P\left({new}|{event}\right)-P\left({reference}|{event}\right)$$

11 $${{IDI}}_{{non}-{event}}=P\left({reference}|{nonevent}\right)-P\left({new}|{nonevent}\right)$$

12 $${{IDI}={IDI}}_{{event}}+{{IDI}}_{{non}-{event}}$$

Risk Assessment Plots provide visual depiction of NRI and IDI reclassification metrics (Fig. 5).

### Association of HE with poor outcomes and death

To evaluate the clinical relevance of HE prediction, we tested the association of model predictions with 3-month clinical outcome. Similar to prior studies^27^, we defined poor functional outcome by mRS score 4–6 at 3-month follow-up. In the independent test cohort, we separately determined the odds ratios of 3-month follow-up poor outcomes as well as death during follow-up period in patients who had HE (ground truth) as well as those predicted to have HE by the deep-learning models.

### Supplementary information

Supplementary material

## Data Availability

The datasets generated and/or analyzed during the current study are available from the corresponding authors (Kevin. N Sheth OR Seyedmehdi Payabvash) upon reasonable request.

## Code availability

The source code is available at the following GitHub repository: https://github.com/anhtrnyaleedu/HE.
