# Supplementary material for: Uncertainty-aware deep-learning model for prediction of supratentorial hematoma expansion from admission non-contrast head computed tomography scan
Source: NPJ Digit Med. 2024 Feb 6;7:26. doi: 10.1038/s41746-024-01007-w (PMC10847454; doi:10.1038/s41746-024-01007-w)
Supplement: Supplementary file 1 — Supplementary material [file 41746_2024_1007_MOESM1_ESM.pdf]

Supplementary Material

1 Supplementary Table 1. Visual predictors of hematoma expansion (HE) on non-contrast head CT........2

2 Supplementary Table 2. Demographic and clinical characteristics of patients in ATACH-2 and Yale datasets. 3

3 Supplementary Table 3. Confusion matrix for prediction of hematoma expansion (HE).4

4 Supplementary Table 4. Performance metrics of the deep learning models for prediction of hematoma expansion (HE) with certainty 5

5 Supplementary Table 5. Visual predictors of hematoma expansion (HE) in training/cross-validation versus the test set 6

6 Supplementary Figure 1. Example head CT slices and manually segmented hematoma lesion mask........7

7 Supplementary Figure 2. The structure of proposed method CNN model for HE prediction 8

8 Supplementary Figure 3. Example axial slices and dilated masks used for model input..9

9 Supplementary Figure 4. Example data augmentation process for axial slices and dilated masks inputs for the CNN model........10

10 Supplementary Figure 5. Example of validation fold AUC and loss diagram during training/cross-validation 11

11 Supplementary Figure 6. ROC curves of the proposed model for $HE_{\geq 6mL}$ prediction in cross-validation folds........12

12 Supplementary Figure 7. ROC curves of the proposed model for $HE_{\geq 3mL}$ prediction in cross-validation folds........13

13 References........14

Supplementary Table 1. Visual predictors of hematoma expansion (HE) on non-contrast head CT

Description of the non-contrast head CT markers of hematoma expansion (HE)	
Blend sign[1]	Blending of relatively hypoattenuating area with adjacent hyperattenuating region within the hematoma with a well-defined margin between these two regions and >18 Hounsfield Unit (HU) difference between the two regions
Swirl sign[2]	Region(s) of hypoattenuation or isoattenuation (compared to brain parenchyma) within the hyperattenuating hematoma. The areas of hypoattenuation or isoattenuation may vary in shape and can be rounded, streak-like or irregular
Black hole sign[3]	Hypoattenuating are encapsulated within the hyperattenuating hematoma with a clearly defined border, and >28 HU difference between the two density regions
Island sign[4]	Either (1) ≥3 scattered small hematomas all separate from the main hematoma or (2) ≥4 small hematomas some or all of which may connect with the main hematoma
Satellite sign[5]	Presence of a small hematoma (< 10mm) separated (by 1–20 mm) from the main hemorrhage on at least one slice
Fluid level sign[6]	Clear horizontal line separating lower from higher density hemorrhage in the dependent aspect of hematoma
Irregular shape sign[7]	Presence of at least two connected or separated irregular hemorrhages at the edge of the hematoma on axial slices
Hypodensity sign[8]	Any hypodense region strictly encapsulated within the hematoma of any shape, size, and density without specific HU difference

2 **Supplementary Table 2. Demographic and clinical characteristics of patients in ATACH-2 and Yale datasets.**

	**ATACH-2 (n=610)**	**Yale (n=183)**	**P value**
**Hematoma expansion ≥6mL** – n (%)	96 (15.7 %)	34 (18.6 %)	0.093
**Hematoma expansion ≥3mL** – n (%)	153 (25.1 %)	48 (26.2 %)	0.754
**Sex** [male] – n (%)	366 (60.0 %)	94 (51.4 %)	0.027
**Age*** [years] – mean ± SD	62.2 ± 13.0	67.5 ± 16.2	0.346
**Ethnic group** – n (%)			
Hispanic	65 (10.6 %)	13 (7.1 %)	0.157
Not Hispanic	545 (89.4 %)	170 (92.9 %)	
**Race** – n (%)			
White	244 (40.0 %)	138 (75.4 %)	<0.001
Black	111 (18.2 %)	30 (16.4 %)	0.576
Asian	239 (39.2 %)	6 (3.3 %)	<0.001
Other	16 (2.6%)	9 (4.9 %)	
**Systolic blood pressure*** [mmHg] mean ± SD	171.6 ± 25.0	170.5 ± 33.1	0.158
**History of hypertension** – n (%)	494 (80.9 %)	144 (78.6 %)	0.492
**History of diabetes mellitus types I/II** – n (%)	146 (23.9 %)	39 (21.3 %)	0.462
**History of hyperlipidemia** – n (%)	204 (33.4 %)	85 (46.4 %)	<0.001
**History of atrial fibrillation** – n (%)	35 (5.7 %)	42 (9.5 %)	0.182
**Glasgow Coma Sca score at baseline** – n (%)			
3-11	85 (13.9 %)	39 (21.3 %)	
12-14	170 (27.8 %)	50 (27.4 %)	0.182
15	356 (58.3 %)	84 (45.9 %)	
unknown	1 (0.1 %)	0	
**NIH Stroke Scale score at baseline** – n (%)			
0-4	101 (16.5 %)	76 (41.5 %)	
5-9	157 (25.7 %)	29 (15.8 %)	
10-14	157 (25.7 %)	30 (16.4 %)	
15-19	108 (17.7 %)	23 (12.4 %)	0.008
20-25	71 (11.6 %)	16 (8.6 %)	
>25	13 (3.1 %)	9 (4.7 %)	
unknown	2 (0.4 %)	0	
**Baseline hematoma volume*** [mL] – mean ± SD	13.12 ± 12.88	23.19 ± 23.46	<0.001
**Follow-up hematoma volume*** [mL] – mean ± SD	16.16 ± 17.86	24.57 ± 23.95	<0.001
**CT**			
Slice thickness* [mm] – mean ± SD	4.6 ± 1.0	5.08 ± 0.42	
Min axial image matrix [n x n]	462 x 462	512 x 512	
Max axial matrix [n x n]	512 x 708	512 x 717	
Number of slices – mean ± SD	35.9 ± 17.5	34.2 ± 5.8	

*Using two-sample t-tests; others using the chi-square test

3 **Supplementary Table 3. Confusion matrix for prediction of hematoma expansion (HE)**

HE prediction based on a threshold with the best F1-score (a harmonic mean of precision and recall)						
**≥6 mL HE**				**≥3 mL HE**		
**Ground truth**	**Model prediction**			**Ground truth**	**Model prediction**	
	HE –	HE +			HE –	HE +
HE –	115	12		HE –	92	18
HE +	9	15		HE +	13	20

HE prediction based on a threshold with higher weighted F score for balanced sensitivity, specificity, and accuracy						
**≥6 mL HE (F2.3)**				**≥3 mL HE (F1.3)**		
**Ground truth**	**Model prediction**			**Ground truth**	**Model prediction**	
	HE –	HE +			HE –	HE +
HE –	103	24		HE –	92	18
HE +	6	18		HE +	13	20

4 **Supplementary Table 4. Performance metrics of the deep learning models for prediction of hematoma expansion (HE) with certainty**

≥6 mL HE	Precision	Recall	F1-score	Patients
**HE –**	0.94	0.81	0.87	127
**HE +**	0.43	0.75	0.55	24
		**Accuracy**	0.80	151 (total)
		**Matthew's correlation coefficient**	0.46	
**≥3 mL HE**	**Precision**	**Recall**	**F1-score**	**Patients**
**HE –**	0.95	0.49	0.65	110
**HE +**	0.35	0.91	0.50	33
		**Accuracy**	0.59	143 (total)
		**Matthew's correlation coefficient**	0.34	

5 Supplementary Table 5. Visual predictors of hematoma expansion (HE) in training/cross-validation versus the test set

Visual predictors of HE – n (%)	Data split for prediction		
	Training/cross-validation (n=634)	Independent testing (n=159)	P value
Blend sign	157 (24.7%)	29 (18.2%)	<0.001
Swirl sign	145 (22.9%)	38 (23.9%)	<0.001
Black hole sign	153 (24.1%)	30 (18.9%)	0.006
Island sign	71 (11.2%)	13 (8.2%)	<0.001
Satellite sign	168 (26.5%)	42 (26.4%)	0.012
Fluid level sign	12 (1.9%)	4 (2.5%)	0.036
Irregular shape sign	283 (44.6%)	59 (37.1%)	<0.001
Hypodensity sign	102 (16.1%)	28 (17.6%)	<0.001

p-value is computed using Fisher extract test.

6 Supplementary Figure 1. Example head CT slices and manually segmented hematoma lesion mask

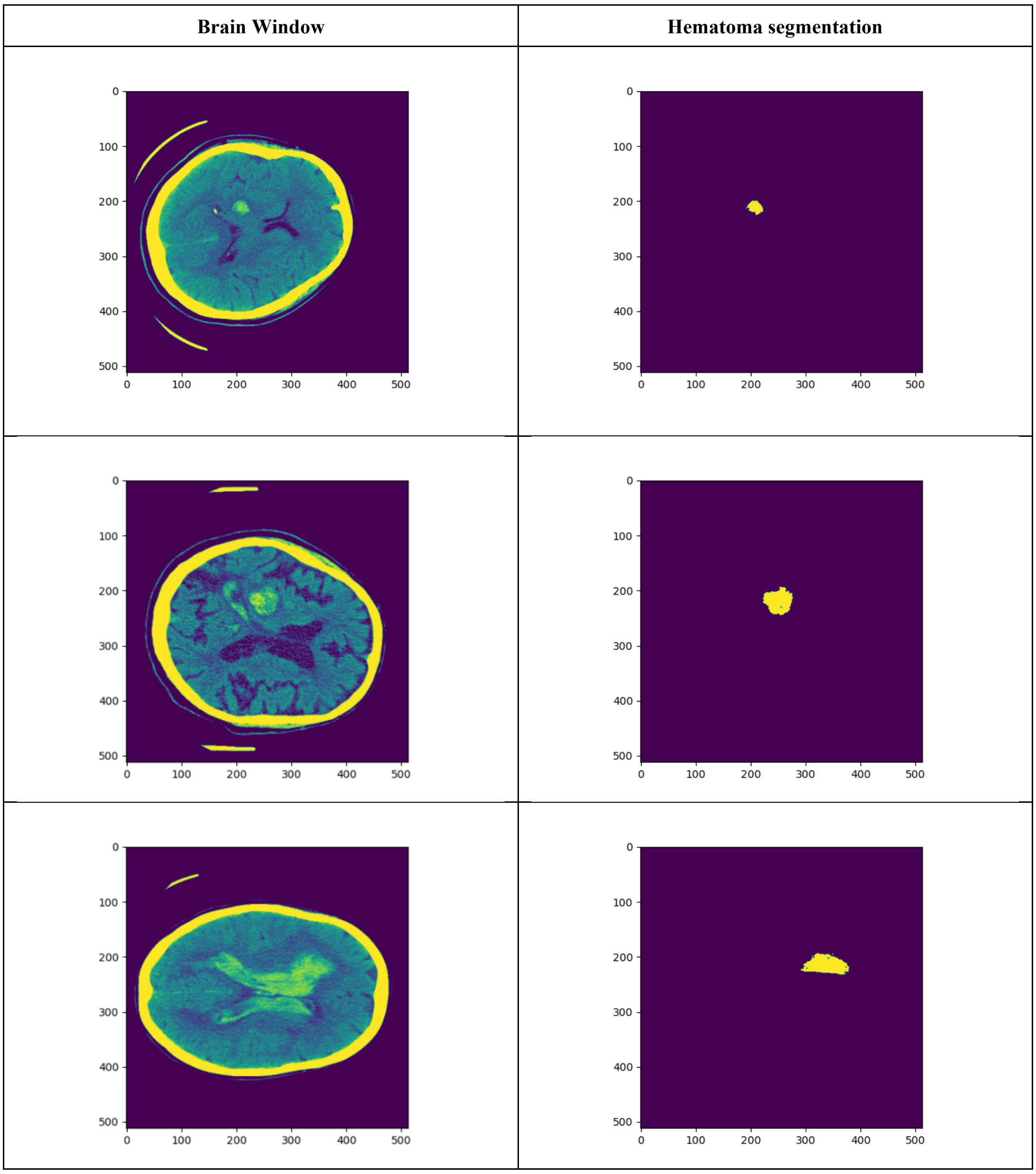

7 Supplementary Figure 2. The structure of proposed method CNN model for HE prediction

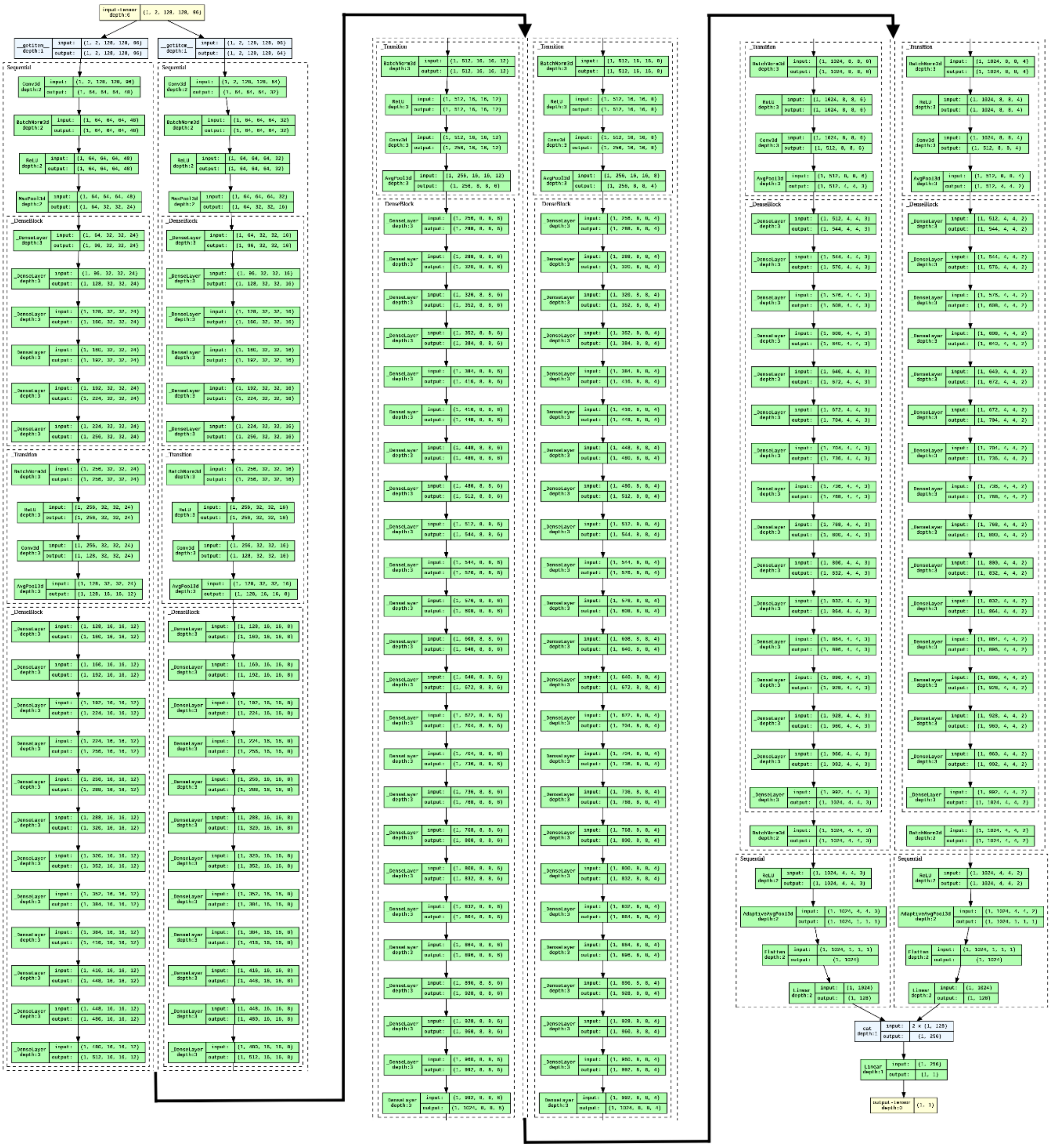

The code of the model and graph are available on GitHub:

https://github.com/anhtrnyaleedu/HE/blob/main/MyDenseNet.py

https://github.com/anhtrnyaleedu/HE/blob/main/model_summary_graph.png

8 Supplementary Figure 3. Example axial slices and dilated masks used for model input

Example slices in 3D input (2, 192, 192, 96)

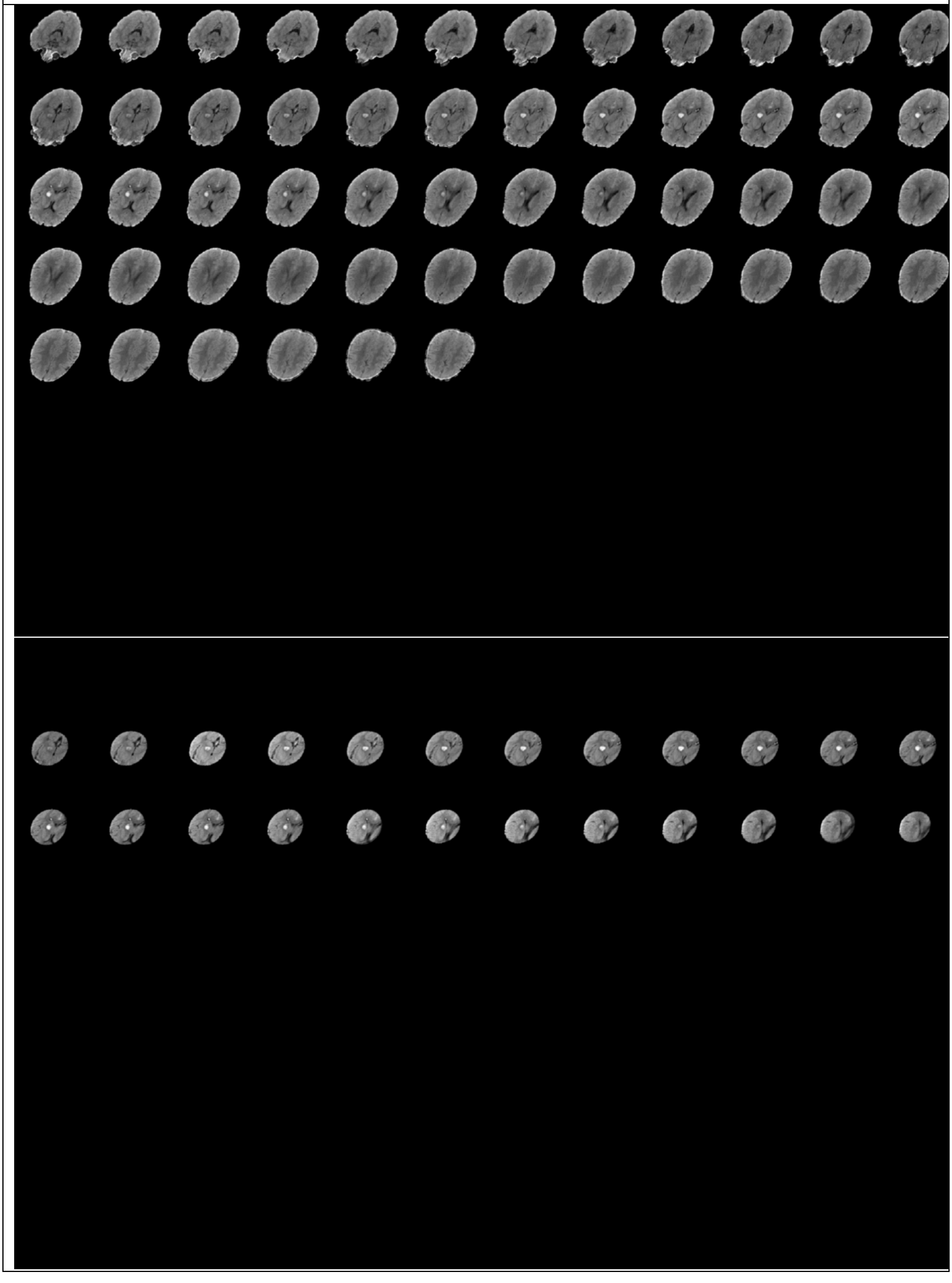

9 Supplementary Figure 4. Example data augmentation process for axial slices and dilated masks inputs for the CNN model

Example augmentation using RandZoom(), RandRotate(), RandAffine(), RandFlip()

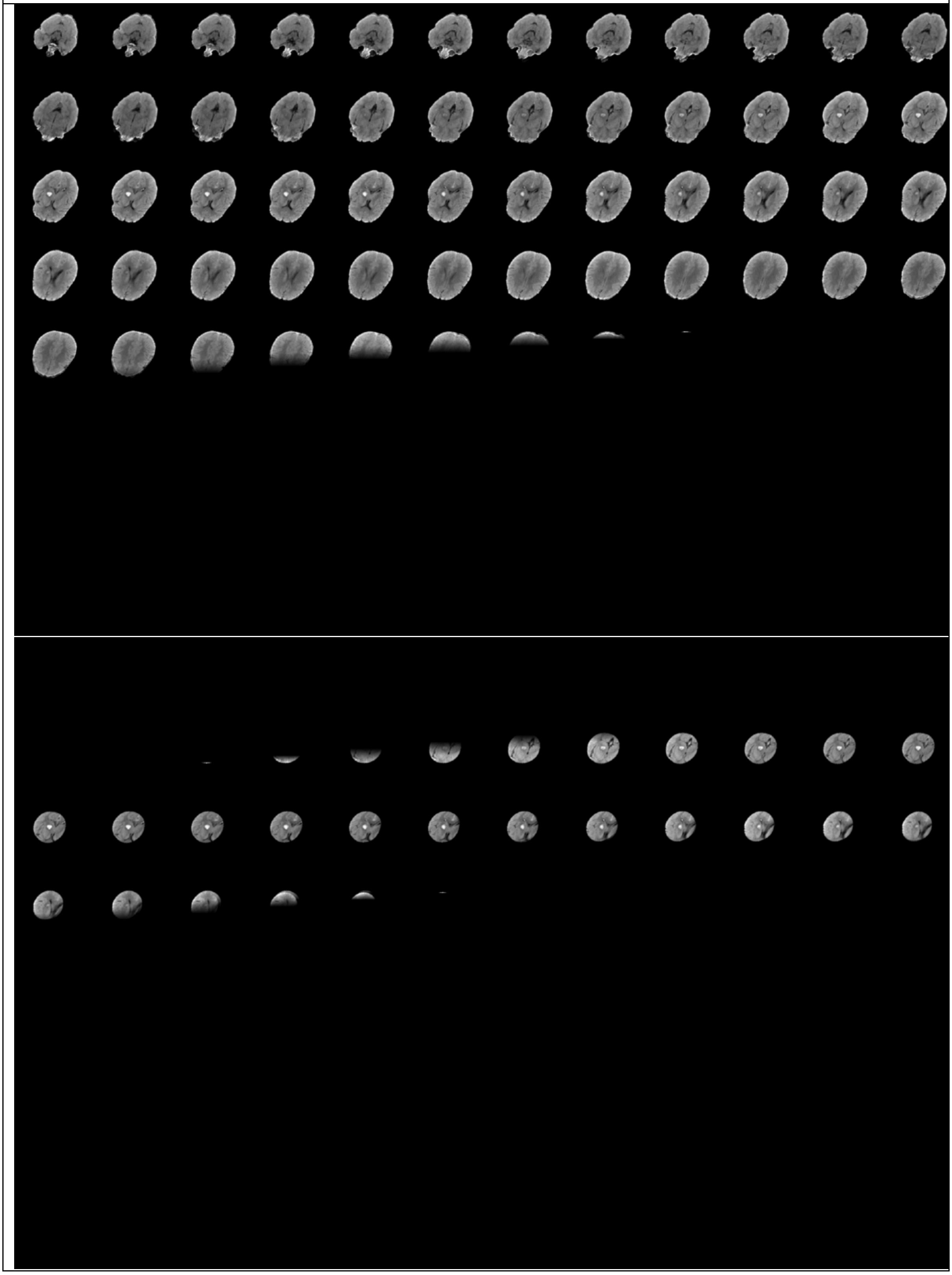

10 Supplementary Figure 5. Example of validation fold AUC and loss diagram during training/cross-validation

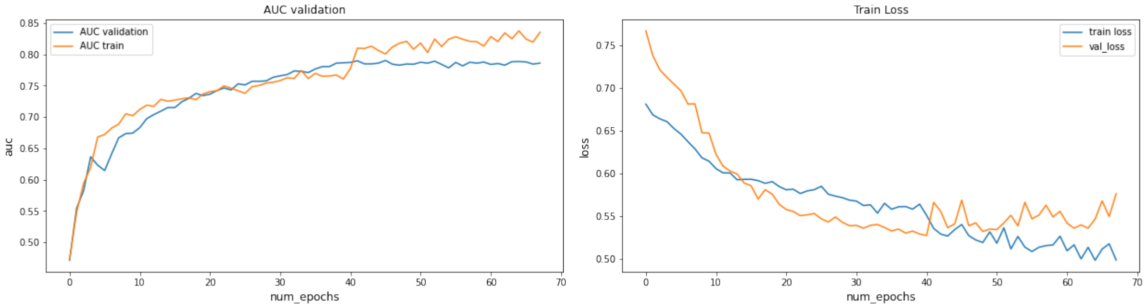

11 Supplementary Figure 6. ROC curves of the proposed model for $HE_{\geq 6mL}$ prediction in cross-validation folds

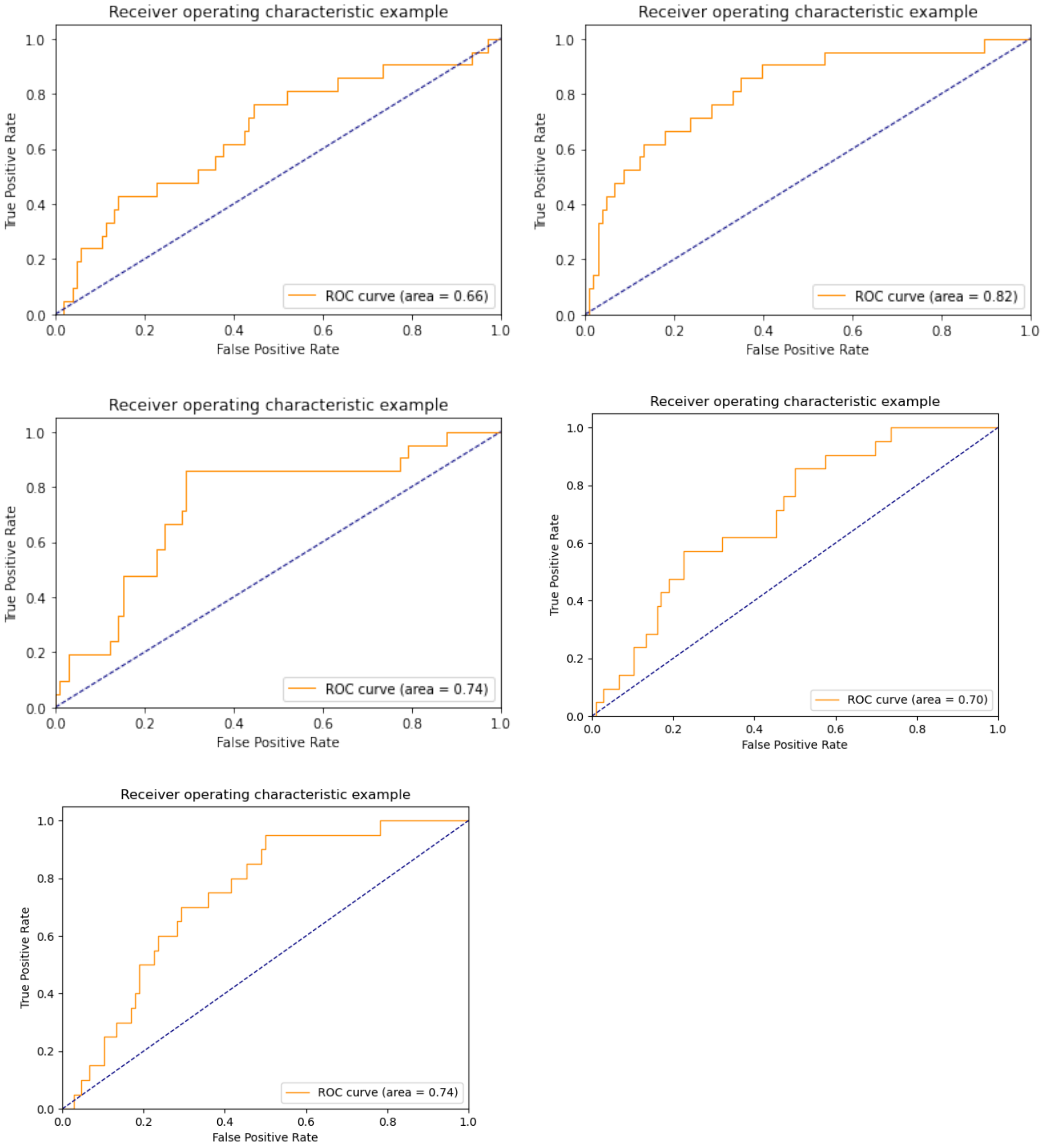

12 Supplementary Figure 7. ROC curves of the proposed model for $HE_{\geq 3mL}$ prediction in cross-validation folds

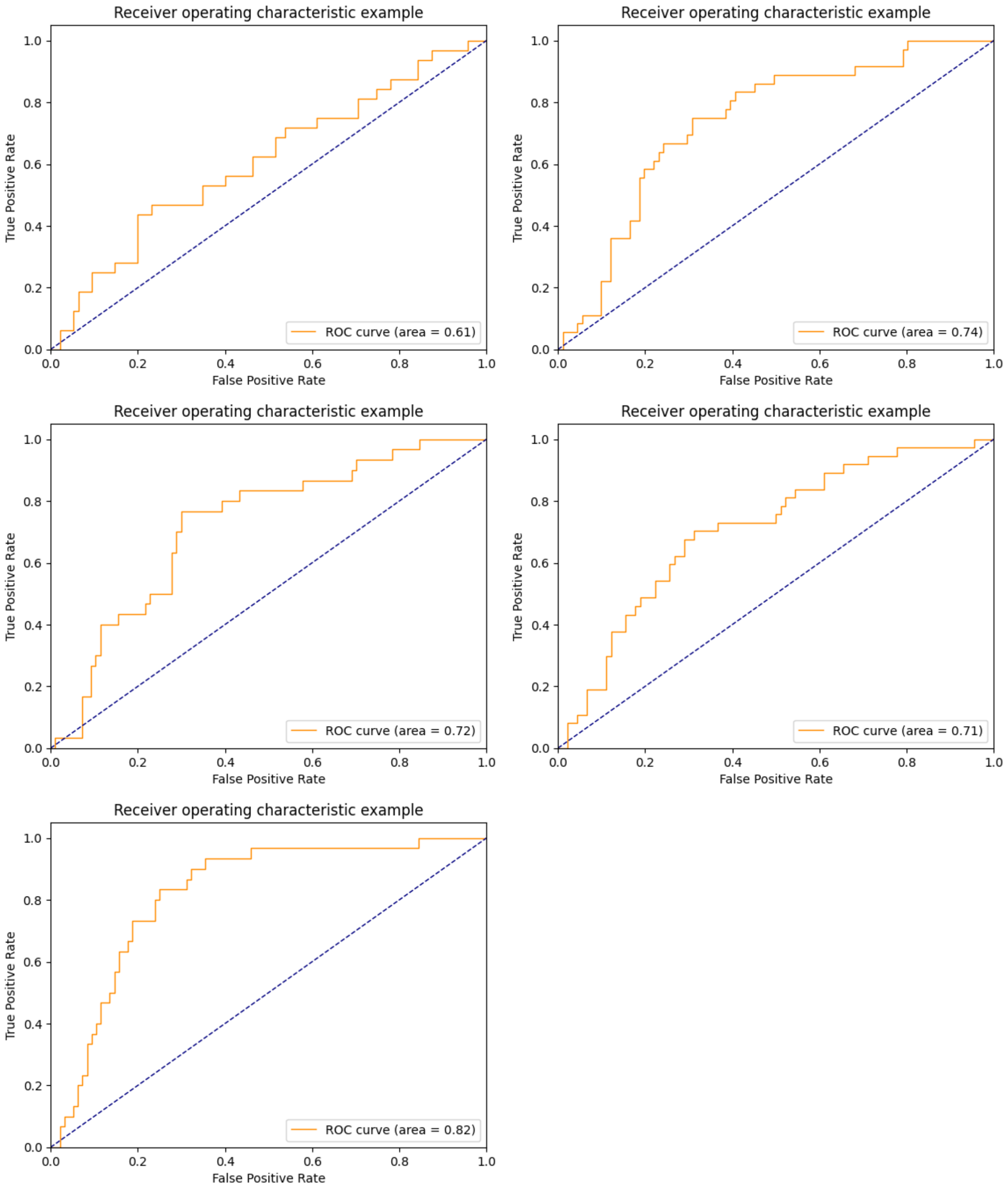

13 References

1 Li Q, Z. G., Huang YJ, Dong MX, Lv FJ, Wei X, Chen JJ, Zhang LJ, Qin XY, Xie P. Blend Sign on Computed Tomography: Novel and Reliable Predictor for Early Hematoma Growth in Patients With Intracerebral Hemorrhage. *Stroke* **46**, 2119–2123 (2015). https://doi.org/10.1161/STROKEAHA.115.009185

2 J. Kim, A. S., J.C. Hemphill, W.S. Smith, Y. Lu, W.P. Dillon, M. Wintermark. Contrast Extravasation on CT Predicts Mortality in Primary Intracerebral Hemorrhage. *American Journal of Neuroradiology* **29**, 520-525 (2008). https://doi.org/10.3174/ajnr.A0859

3 Qi Li, G. Z., Xin Xiong, Xing-Chen Wang, Wen-Song Yang, Ke-Wei Li, Xiao Wei, Peng Xie. Novel Imaging Marker That Predicts Hematoma Growth in Patients With Intracerebral Hemorrhage. *Stroke* **47**, 1777–1781 (2016). https://doi.org/10.1161/STROKEAHA.116.013186

4 Qi Li, Q.-J. L., Wen-Song Yang, Xing-Chen Wang, Li-Bo Zhao, Xin Xiong, Rui Li, Du Cao, Dan Zhu, Xiao Wei, Peng Xie. An Imaging Predictor for Early Hematoma Expansion and Poor Outcome in Patients With Intracerebral Hemorrhage. *Stroke* **48**, 3019–3025 (2017). https://doi.org/10.1161/STROKEAHA.117.017985

5 Shimoda Y, O. S., Arai H, Okada K, Tominaga T. Satellite Sign: A Poor Outcome Predictor in Intracerebral Hemorrhage. *Cerebrovasc Dis.* **44**, 105-112 (2017). https://doi.org/10.1159/000477179

6 Blacquiere D, D. A., Al-Hazzaa M, Deshpande A, Petrcich W, Aviv RI, Rodriguez-Luna D, Molina CA, Silva Blas Y, Dzialowski I, Czlonkowska A, Boulanger JM, Lum C, Gubitz G, Padma V, Roy J, Kase CS, Bhatia R, Hill MD, Dowlatshahi D. Intracerebral Hematoma Morphologic Appearance on Noncontrast Computed Tomography Predicts Significant Hematoma Expansion. *Stroke* **46**, 3111-3116 (2015). https://doi.org/10.1161/STROKEAHA.115.010566

7 Liu C, Z. H., Wang L, Jiang Q, Lu E, Yuan C, Liang Y, Sun Z, Xiang H, Xu X, Sun J, Fu B, Zhao B, Zhang D, Chen X, Wang N, Wang L, Yang G. Irregular shape as an independent predictor of prognosis in patients with primary intracerebral hemorrhage. *Sci Rep* **12**, 8552 (2022). https://doi.org/10.1038/s41598-022-12536-3

8 Boulouis G, M. A., Brouwers HB, Charidimou A, Jessel MJ, Auriel E, Pontes-Neto O, Ayres A, Vashkevich A, Schwab KM, Rosand J, Viswanathan A, Gurol ME, Greenberg SM, Goldstein JN. Association Between Hypodensities Detected by Computed Tomography and Hematoma Expansion in Patients With Intracerebral Hemorrhage. *JAMA Neurol.* **73**, 961-968 (2016). https://doi.org/10.1001/jamaneurol.2016.1218.
